# Investigation of GOx Stability in a Chitosan Matrix: Applications for Enzymatic Electrodes

**DOI:** 10.3390/s23010465

**Published:** 2023-01-01

**Authors:** Ayman Chmayssem, Ibrahim Shalayel, Stéphane Marinesco, Abdelkader Zebda

**Affiliations:** 1 Université Grenoble Alpes, CNRS, UMR 5525, VetAgro Sup, Grenoble INP, INSERM, TIMC, 38000 Grenoble, France; 2 Plate-Forme Technologique BELIV, Lyon Neuroscience Research Center, CNRS UMR5292, INSERM U1028, Université Claude Bernard Lyon 1, 69373 Lyon, France

**Keywords:** glucose biosensor, chitosan matrix, screen-printed electrode, glucose oxidase (GOx), glutaraldehyde crosslinker

## Abstract

In this study, we designed a new biosensing membrane for the development of an electrochemical glucose biosensor. To proceed, we used a chitosan-based hydrogel that entraps glucose oxidase enzyme (GOx), and we crosslinked the whole matrix using glutaraldehyde, which is known for its quick and reactive crosslinking behavior. Then, the stability of the designed biosensors was investigated over time, according to different storage conditions (in PBS solution at temperatures of 4 °C and 37 °C and in the presence or absence of glucose). In some specific conditions, we found that our biosensor is capable of maintaining its stability for more than six months of storage. We also included catalase to protect the biosensing membranes from the enzymatic reaction by-products (e.g., hydrogen peroxide). This design protects the biocatalytic activity of GOx and enhances the lifetime of the biosensor.

## 1. Introduction

Electrochemical enzymatic glucose biosensors are the most commonly used devices to monitor blood glucose levels in diabetic patients [1,2,3]. Some of these devices can be implanted subcutaneously in diabetic patients to monitor glucose continuously for up to several weeks, and are called continuous glucose monitoring systems (CGMs). They often contain an electrode and a porous polymer, or hydrogel, encapsulating an enzyme, such as glucose oxidase (GOx), that catalyzes glucose oxidation.

However, commercially available biosensors suffer from low stability for long-time operation and a gradual decrease in sensitivity with storage. Due to the enzyme’s fragility, enzyme-based biosensors typically show a loss of sensitivity over time and consequently, express short lifetime. The loss of sensitivity implies either a replacement or a regular calibration of the sensors [4,5]. Biosensor lifetime is also limited by inflammatory reactions, or foreign body reactions that isolate the device from healthy interstitial fluid. However, providing sensors with enhanced lifetime would improve patient’s quality of life [6,7]. Therefore, it is interesting to develop new methods for stabilizing the enzyme and enhancing glucose biosensors lifetime.

Several strategies have been proposed to enhance enzymatic biosensor lifetime, such as enzyme immobilization and enzyme engineering [8,9]. Indeed, the first strategy remains the most popular, due to its simplicity. There are different enzyme immobilization techniques, including physical adsorption, surface binding by covalent and enzyme encapsulation, and/or entrapment in polymeric matrix [10,11]. In recent years, enzyme encapsulation in hydrogel or polymers has attracted great interest because they adsorb water and provide a microenvironment favorable for enzyme stability over time. However, physical and chemical proprieties of these matrixes (i.e., surface area and roughness, pores size and distribution, membrane thickness and composition, etc.) play a key role in the stability of encapsulated or entrapped enzymes [12]. Depending on those properties, diffusion of glucose through membranes and then the enzyme accessibility may be affected, which influences the biocatalytic activity of the enzymes. Therefore, the choice of polymers or hydrogel-based matrixes and their nature and composition are of great importance.

Many materials were reported as potential candidates for the development of biomembranes such as cellulose [13,14], polycarbonate [15], hyaluronic acid [16], collagen [17,18] and chitosan [19,20]. However, chitosan-based materials are of particular interest due to their particular structural and functional characteristics, i.e., biocompatibility, high permeability, antibacterial and antimicrobial properties, adsorptive characteristics, and low cost [21]. Minteer et al. suggested that enzyme encapsulation within the pores/pockets of the hydrophobically modified micellar polymers, such as Nafion^®^ and chitosan, drastically improves enzymes lifetime [22]. S El ICHI et al. reported that laccase encapsulation in chitosan multi-walled carbon nanotubes composite allows designing a biocathode with several months’ stability [23,24]. Gorski et al. showed that modifying chitosan with a permeability-controlling agent, Acetyl Yellow 9 (AY9), decreases the sensing potential and allows for the interference-free determination of the enzyme’s substrate [25,26]. He also demonstrated in a different work that chitosan could be incorporated in a carbon nanotube powder allowing for the designing of a second-generation biosensor using different enzyme substrates [27,28]. Similarly, in a previous work, we investigated the stability of glucose dehydrogenase (GDH) in a chitosan-MWCNTs pellet and showed enhanced GDH stability for more than one year [29]. In these studies, the introduction of crosslinkers has been proposed to enhance chitosan stability. Indeed, chitosan is a biodegradable polymer that suffers from a loss of stability in an aqueous solution over time, which requires the use of a crosslinker such as genipin or glutaraldehyde. Conversely to genipin, the crosslinking process of chitosan by glutaraldehyde does not demand heating, and can be achieved at room temperature, which prevents enzyme denaturation [30,31]. This makes glutaraldehyde an option of choice for the development of a chitosan-based biomembrane.

In the case of first-generation enzymatic glucose biosensor where the enzymatic oxidation of glucose by GOx leads to the production hydrogen peroxide (H_2_O_2_) and gluconic acid, the chitosan matrix should be capable of maintaining its stability in the presence of H_2_O_2_ and in an acidic microenvironment resulting from the accumulation of gluconic acid. The aim of the present work is to design a chitosan-based enzymatic glucose biosensor and the study of its stability over time. Our strategy consisted of crosslinking and encapsulating the enzyme GOx into/with the chitosan matrix. Here, we investigated the adhesion of chitosan on the surface of different electrode materials (gold, platinum, and carbon). The stability of the biosensors over time according to different storage conditions (in PBS at 4 °C/37 °C and in the presence or absence of glucose) was also investigated. We also investigated the stability of chitosan-based membranes under dry storage conditions at various temperatures of 4 °C, 37 °C, and at room temperature.

## 2. Materials and Methods

### 2.1. Chemicals

Glutaraldehyde solution—grade 1, 70% in water (C_5_H_8_O_2_, CAS No: 111-30-8, ref. G7776), Thiol-NH_2_ powder (HS-PEG2K-NH_2_, ref. JKA5143), collagen from bovine Achilles tendon (CAS No: 9007-34-5, ref. C9879), chitosan high molecular weight (310–375 kDa, >75% deacetylated, CAS No: 9007-34-5, ref. 419419), D-Glucose powder (purity > 99.5%, CAS No: 50-99-7, ref. G8270), GOx from Aspergillus Niger (type X-S lyophilized powder, CAS No: 9001-37-0, ref. G7141-50KU), GOx from Aspergillus niger recombinant (CAS No: 9001-37-0, ref. 345386-10KU) and catalase from bovine liver (CAS No: 9001-05-2, ref. C1345) were purchased from Sigma-Aldrich. Stock solution of phosphate-buffered saline (PBS 10x) was also purchased from Sigma-Aldrich and utilized for the preparation of the working solutions (PBS 1x pH 7.4). All reagents were of analytical grade, and milli-Q water was used to prepare solutions.

### 2.2. Preparation of Hydrogels

Collagen solution (1 wt%) was prepared by dissolving collagen powder in acetic acid solution (0.1 *v*/*v* %). The obtained emulsion was stirred at room temperature for 24 h until the collagen powder was fully dissolved. The obtained gel was stored in the fridge at 4 °C when not in use.

Chitosan solution (1 wt%) was prepared by dissolving chitosan powder (high molecular weight) in acetic acid solution (0.5 *v*/*v* %). The mixture was left to stir at 70 °C for 1 h to fully dissolve the chitosan. The obtained gel was then placed at room temperature to cool down, and stored in the fridge at 4 °C when not in use. The 1% concentration of chitosan was chosen to limit acetic acid accumulation as much as possible. A higher concentration of acetic acid is required to dissolve chitosan powder when the chitosan concentration increases.

### 2.3. Electrochemical Apparatus and Electrodes

An SP-300 potentiostat (Biologic Instruments, Grenoble–France) was used for all electrochemical experiments. Screen-printed gold electrodes (auxiliary electrode: Pt and reference electrode: Ag, ref. DRP-250AT-U75) were purchased from METROHM (Villebon-sur-Yvette, France). The working electrodes have a 4 mm diameter characteristic which represents a surface area of 12.56 mm^2^.

### 2.4. Electrode Design

For the chitosan-based materials, it was necessary to crosslink the chitosan-hydrogel by adding glutaraldehyde in order to ensure its stability. Our strategy consisted of combining crosslinking and encapsulating the GOx into/within the chitosan matrix. This allows for large amounts of enzymes to be immobilized and stabilizes the GOx enzyme being used [32]. Indeed, some trials conducted in our lab showed that the chitosan matrixes require the use of an adhesion layer specifically dedicated to the commercial gold-based electrodes (METROHM, DRP-250AT). For this purpose, a collagen solution (1 wt%) was used in the presence of glutaraldehyde (added at 2 wt% vs. collagen) and mixed. Five minutes later, 10 µL of the mixture was pipetted and deposed by drop-casting on the working Au-based electrodes and maintained at room temperature for complete drying (approx. 1 h) to provide an intermediate layer that avoided the shrinkage of the chitosan matrix on the working Au-based electrodes [33].

**Figure 1:**
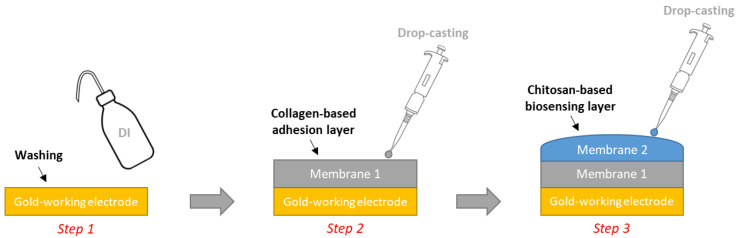
Illustration of the modification process of the working gold-based screen-printed electrode for the fabrication of glucose biosensors.

For the preparation of the chitosan matrix: 10 mg of GOx was added to 1 mL chitosan solution (1 wt%), and the mixture was gently shaken for a few seconds (IKA^®^ Vortex). Glutaraldehyde solution (2 wt% related to chitosan) was then added after 5 min, and 10 µL from the prepared mixture was deposed by drop-casting as a second layer on the collagen layer. The electrodes were kept at room temperature until complete drying was achieved (approx. 1 h). Finally, the sensors were rinsed with distilled water (Figure 1).

### 2.5. Stability Test of Biosensing Membranes

We investigated the stability of the GOx-chitosan-based glucose biosensor under different storage conditions. The biosensors were first tested in PBS (1X) medium by chronoamperometry. Thereby, the sensors were polarized at + 0.7 V vs. Ag/AgCl via the reference electrode (Figure S1). The electrochemical response was then recorded in presence of an increasing concentration of glucose in the bulk solution under a dynamic condition of 200 rpm. This allows for the establishment of the calibration curve (i_glucose_ = f ([Glucose]) and the determination of the sensitivity of the biosensors at the first day. Herein, the sensitivity corresponds to the slope value of the calibration curve (expressed in nA/mM).

## 3. Results and Discussion

**Figure 2:**
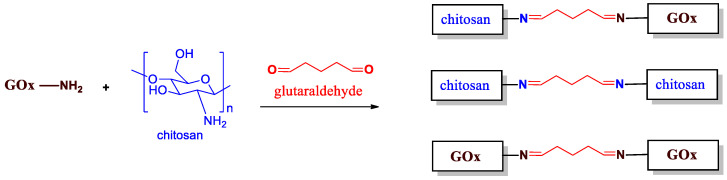
Immobilization process of the enzyme GOx into the chitosan polymer using the glutaraldehyde crosslinker (added at 2 wt%).

In order to highlight the immobilization process, it was necessary to understand the reaction that could occur during the fabrication of the bio-based membranes. Indeed, the Gox, as well as the chitosan polymer, are well-known to contain many amine groups in their structures. This feature facilitates the derivatization process. Thus, in the presence of the glutaraldehyde crosslinker (added at 2 wt%), a non-selective reaction leads to obtain three reaction byproducts, as represented in Figure 2. The best scenario is reached when the chitosan is crosslinked and bonded to the GOx enzyme through the glutaraldehyde chain. This configuration specifically prevents from enzyme’s release from the chitosan matrix into solution. However, a chitosan polymer could be crosslinked to another chitosan polymer chain, and the GOx could be also crosslinked and bonded to another GOx enzyme through a glutaraldehyde chain. This reaction occurs randomly; thus, the homogeneity of the GOx/chitosan mixture is of great importance in order to obtain reproducible results.

**Figure 3:**
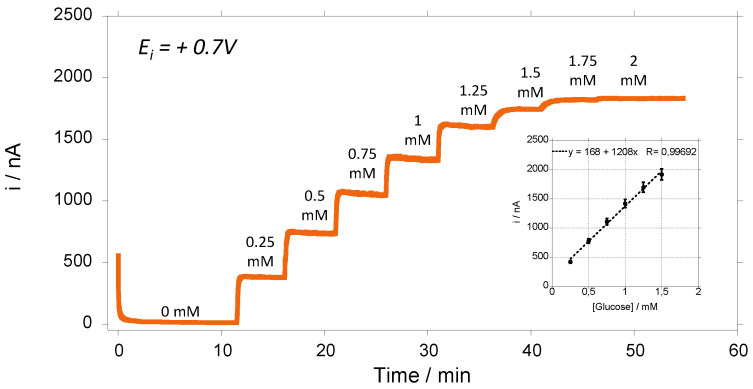
Amperometric responses at + 0.7 V vs. Ag/AgCl of glucose biosensing) in PBS(1X) medium under hydrodynamic conditions of 200 rpm. Inset: calibration curve of glucose during electrochemical oxidation (average value and standard deviation on 3 electrodes).

Figure 3 shows the amperometric response of GOx-chitosan biosensor to successive additions of glucose. We can see that we have a typical amperometric response as a function of glucose concentration showed a linear relationship (r^2^ > 0.996) in a concentration range of glucose of 0.25–1.5 mM (Figure 3 inset). The apparent Michaelis–Menten constant (K_m_^app^) of the GOx was determined, and it is near 1 mM. Initially, the biosensors were kept and stored in a phosphate buffer solution (PBS 1X) in the absence or presence of glucose (2 mM) in the solution. After that, the biosensors are retested to investigate the variation of their sensitivities at different times. The results are always normalized according to the value of the sensors’ sensitivities obtained at day 1 for each biosensor.

**Figure 4:**
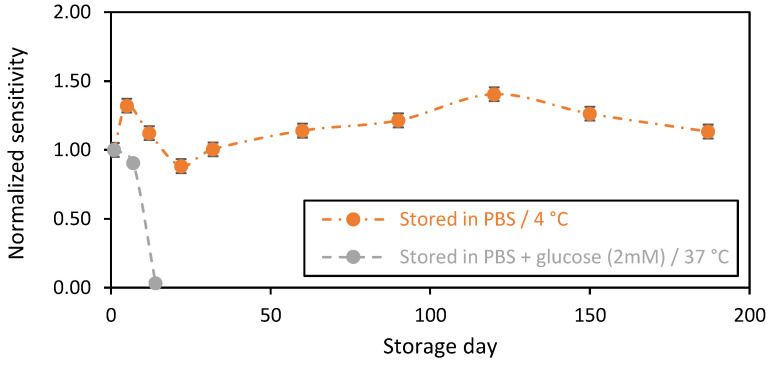
Stability in function of time of the chitosan-based biosensing membranes deposed on gold screen-printed electrodes. Orange plot: biosensors stored in PBS at 4 °C, and grey plot: PBS + glucose (2 mM) at 37 °C (average value and standard deviation on 2 sensors).

The changes in biosensors sensitivity as a function of the storage day in PBS (1X) in the presence of glucose at 2 mM at 37 °C (Figure 4, grey plot) show a stable sensitivity in the first 7 days of storage, with only a 10% decrease in sensitivity after 1 week of storage. The sensitivity drastically decreased after 2 weeks of storage, with a 90% loss of sensitivity (Figure 4), probably due to the degradation of the chitosan-based membrane. Indeed, in the presence of glucose in the storage solution, GOx continuously catalyzes the oxidation of glucose into hydrogen peroxide (H_2_O_2_) and gluconic acid, according to the following reaction [2,34]:

$$Glucose+O_{2}\;\overset{\;GOx\;}{\to }H_{2}O_{2}+gluconic\;acid$$

However, it is well known that the accumulation of the enzymatic reaction by-products, in particular, H_2_O_2_ at high concentration levels, could contribute to the change in the electrode material properties [35,36,37]. Herein, the pH of the storage solution was investigated and followed over time. A slight acidification of the medium was detected (ΔpH ≈ 0.2), despite the presence of phosphate buffer, confirming the accumulation of acidic byproducts of the enzymatic reaction. Thus, this observation could help to confirm our hypothesis. It is important that we note that the pH value can be lower inside the matrix due to a local acidification of the nearby chitosan GOxs sites. Indeed, the activity of the immobilized GOx is pH-dependent. Thus, the local acidification in chitosan matrixes negatively affects the catalytic activity of the GOxs and acts to decrease the sensor’s sensitivities over time [38]. On the other hand, the continuous measurement of glucose for 24 h was studied (Figure S2). We observed a significant drift, compared to that in the storage condition. We believe here that this drift is more related to the degradation of the electrode under H_2_O_2_ electrooxidation, which is capable of passivating the electrode surface. This is a typical behavior of the gold-based sensors (signal drift) that should be considered and corrected. For the biosensors that were stored in PBS without any glucose at 4 °C (orange plot), functional stability was observed for more than 6 months of storage. The changes in the biosensors sensitivities over time may be due to the conditioning of the chitosan-based matrix. For instance, chitosan material is well known for its high swelling capacity, which could affect the enzyme stability over time [39]. Indeed, it is noteworthy to highlight that the apparent Michaelis–Menten constant (K_m_^app^) of the GOx was not affected during the long storage period, and it expressed the same value over time of ~1 mM.

Until now, and for all these samples, GOx and chitosan crosslinking was performed in a single step and that leads to a chitosan matrix where a single GOx may be crosslinked to chitosan. As mentioned above, one step crosslinking leads to the random distribution and crosslinking of GOx. It was interesting to study the effect of the crosslinking order on the stability of the biosensing membranes. To do so, we first crosslinked GOx molecules and subsequently added them to chitosan. In contrast to the first membrane design, where the crosslinking was realized non-selectively between the GOx and the chitosan, herein, only one reaction product was expected, considering that the GOx molecules are crosslinked and bonded together. In the latter design, the GOx molecules are entrapped without any covalent bonds within the chitosan polymer. Here, we assumed that all the glutaraldehyde was consumed because of its high reactivity and thus, we supposed that no more glutaraldehyde was left to react and crosslink the chitosan polymer.

**Figure 5:**
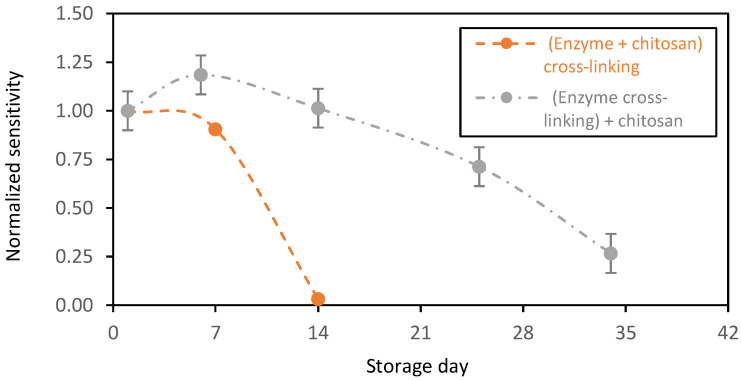
Stability in function of time of the chitosan-based biosensing membranes deposed on gold screen-printed electrodes and stored in PBS + glucose (2 mM) at 37 °C. Orange plot: GOx entrapped and crosslinked, and grey plot: GOx entrapped (average value and standard deviation on 2 sensors).

This new enzyme immobilization design displayed higher stability (Figure 5, grey plot). The biosensors showed an increased normalized sensitivity of ~ 1.18 for the first week of storage in PBS in the presence of glucose at 2 mM (normalized with respect to first day of operation). This is probably due to the swelling capacity of the chitosan matrixes that increased the glucose diffusion to the GOx active sites. Afterwards, the biosensor sensitivity decreased progressively over time, probably due to a decrease in the biocatalytic activity of the GOx. After one month of storage, the sensors expressed a normalized sensitivity near 0.25. During this period, the sensors were stored in the presence of glucose with continuous enzymatic activity. Despite this decrease in the biocatalytic activity of the GOx, this immobilization method stabilized the redox enzyme that was active for more than one month.

We then compared the performance of our GOx-chitosan based glucose biosensors to recombinant GOx (rGOx)-chitosan based glucose biosensors. Indeed, the rGOx is a structurally modified enzyme on a nanoscopic scale. The recombinant enzyme displays similar properties to the native enzyme, but exhibits more advantageous properties for glucose oxidation, including the specificity to the substrate (glucose) and an enhanced stability over time [40,41]. Additionally, rGOx might contain more impurities, including enzymatic impurities. In particular, rGOx contains catalase as an enzymatic impurity at a higher percentage compared to the native GOx from Aspergillus niger. Here, it should be noted that catalase, an oxidoreductase, catalyzes the decomposition of H_2_O_2_ into O_2_ and water as follows [42]:

$$H_{2}O_{2}\;\overset{\;Catalase\;}{\to }\;H_{2}O+O_{2}$$

According to this reaction, H_2_O_2_ produced by the GOx or rGOx should be consumed/decomposed locally by the catalase into O_2_ and H_2_O. It is noteworthy to highlight that this decomposition should be partial to allow for the detection and oxidization of H_2_O_2_ on the surface of the electrode. In contrast, the complete decomposition of H_2_O_2_ leads to the elimination of the electrochemical response related to H_2_O_2_ oxidation.

**Figure 6:**
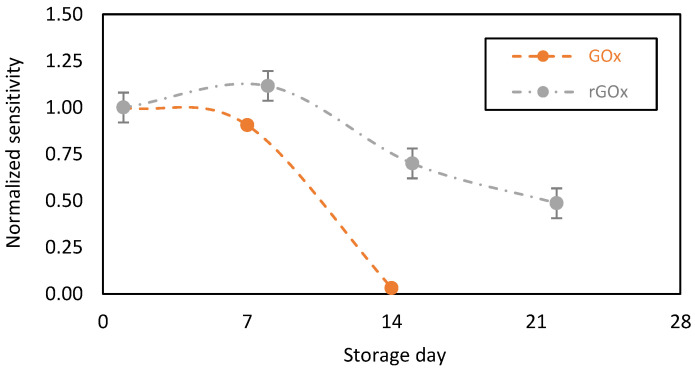
Stability in function of time of the chitosan-based biosensing membranes deposed on gold screen-printed electrodes and stored in PBS + glucose (2 mM) at 37 °C. Orange plot: normal Gox, and grey plot: recombinant rGOx (average value and standard deviation on 2 sensors).

We tested the stability of the biosensing membranes designed with the GOx or rGOx, and found increased rGOx stability over time (Figure 6, grey plot). Nevertheless, after 2 weeks of storage in PBS in the presence of glucose (at 2 mM), a 30% decrease in sensitivity was observed, with a 50% decrease after 3 weeks. This relatively decreases the level of the catalytic current, but it probably protects the chitosan-based membrane from intoxication by the local and continuous accumulation of H_2_O_2_ and prevents the biocatalytic activity of GOx from drastically decreasing.

To verify this hypothesis, we prepared a GOx biosensor in which we added catalase to the native GOx in 2/10 ratio (2 catalase units per 10 GOx units). Each GOx unit oxidizes 1 µmole of glucose, while each unit of catalase theoretically decomposes 1 µmole of H_2_O_2_ per minute. Therefore, mixing catalase with GOx in a 2/10 ratio was expected to decrease the local H_2_O_2_ concentration within the membrane, while preserving glucose detection through H_2_O_2_ reoxidation at the electrode surface.

**Figure 7:**
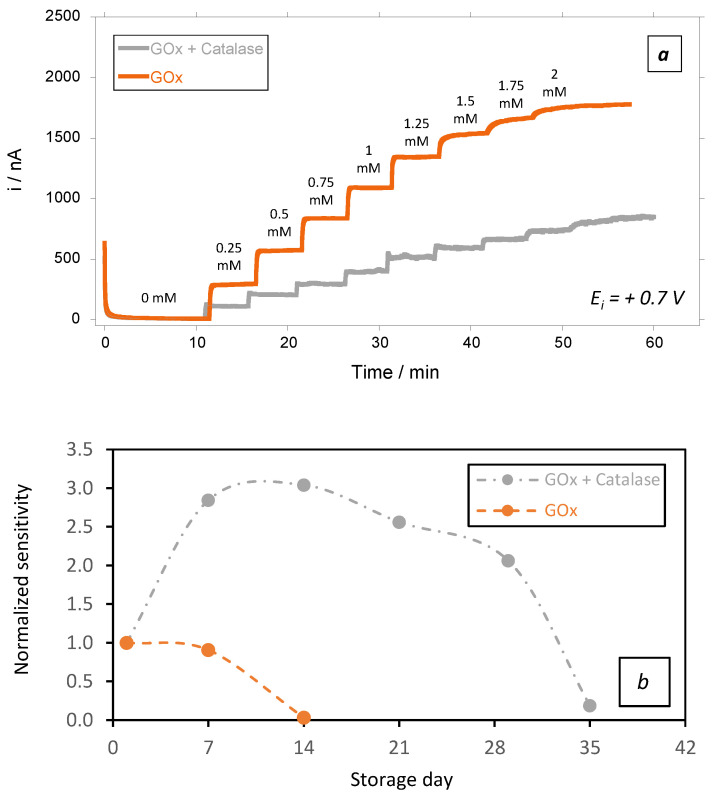
(**a**) Amperometric responses at + 0.7 V vs. Ag/AgCl in PBS(1X) medium under hydrodynamic conditions of 200 rpm for testing of biosensors at day 1 in presence of an increased concentration of glucose, and (**b**) stability in function of time of the chitosan-based biosensing membranes deposed on gold screen-printed electrodes and stored in PBS + glucose (2 mM) at 37 °C: orange plots: GOx, and grey plots: GOx + added catalase (average value and standard deviation on 2 sensors).

Unsurprisingly, the catalytic current observed at day 1 in the presence of catalase was lower than that observed in the absence of catalase (Figure 7a). This could be attributed to the partial decomposition of H_2_O_2_ by catalase, which automatically prevents H_2_O_2_ diffusion to the electrode surface and reduces the catalytic current by cascade. Additionally, the effect of the addition of catalase on the limit of detection (LoD) of the biosensors was also studied. Unsurprisingly, LoD was slightly affected by the presence of catalase (10.8 µM vs. 7.1 µM, respectively, in the absence and in presence of catalase in the biosensing membrane). However, the biosensing membranes containing catalase were also more stable over time (Figure 7b, grey plot). Indeed, even in the presence of glucose (2 mM), there was a remarkable increase in the sensors’ sensitivity that peaked at ~ 3 times its initial sensitivity after 2 weeks of storage. This can be attributed to the swelling capacity of chitosan, as well as to the change in the physicochemical properties of the biomembranes due to the presence of catalase. Indeed, the K_m_^app^ of the GOx was not affected by the presence of catalase. This aspect is of high interest, as it supports that the GOx preserved similar properties in the latter conditions.

These results confirm that the catalase added in the vicinity of GOx firstly, protects the chitosan-based membrane from the intoxication by the locally accumulated H_2_O_2_ and secondly, improves/protects the biocatalytic activity of the GOx.

On the other hand, in addition to the wet storage in PBS(1X) (with or without glucose), it is considerably interesting to investigate the enzymatic biosensor’s stability under dry storage conditions. In fact, for logistical reasons, there is a need to understand the impact of temperature variation on the enzymatic membrane’s stability. For this purpose, the stability of the biosensing membranes was investigated under dry storage conditions at various temperatures of 4 °C (realized in the fridge), 37 °C (realized in the oven), and at room temperature. In the cases where the biosensors were stored at 4 °C and at 37 °C, they maintained stable temperatures. However, for the storage at room temperature, the biosensors suffered from humidity and the change in temperature between day and night. The storage at 4 °C ensures a high humidity level in contrast to the storage in the oven (at 37 °C), where the humidity is relatively low. For all the sensors, the enzymatic reaction is stopped during the dry storage, while the sensors’ sensitivity was evaluated at room temperature by establishing a calibration curve for each sensor and comparing their sensitivities on a normalized scale at different times.

**Figure 8:**
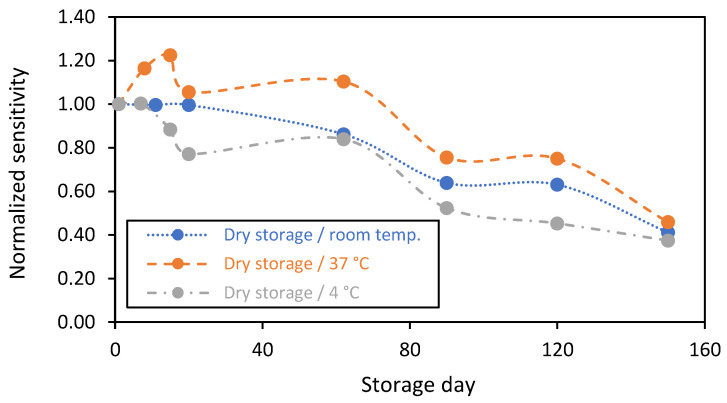
Stability in function of time of the chitosan-based biosensing membranes deposed on gold screen-printed electrodes and stored dry at various temperatures: (blue plot) room temperature, (orange plot) 37 °C, and (grey plot) 4 °C (n = 1 sensor).

The results comparing the stability of the biosensing membranes that were stored dry at the various temperatures for up to 5 months are presented in Figure 8. It is clearly shown that the stability of sensors stored at room temperature (blue plot) was maximized for all the measurements. They expressed stable responses during the first 3 weeks. After that, the sensitivity of the biosensing membranes decreased by 15 and 60% at 2 and 5 months, respectively. However, for the sensors stored at 37 °C (orange plot), the sensitivity increased by 20% after 2 weeks of storage and then fell to 25% and 55% after 3 and 5 months, respectively. The sensors stored at 4 °C (grey plot) showed stable responses in the first week of dry storage, following by a decrease of 23%, 48%, and 63% after 20, 90, and 150 days, respectively. These results confirm that the storage temperature (under dry conditions) impacts the catalytic activity of the GOx and affects the chitosan biomembrane microenvironment. This should be considered in the future for designing long-term in vivo sensor systems.

## 4. Conclusions

A GOx-based first generation glucose biosensor has been successfully designed and its stability over time under different storage and operation conditions has been investigated. Our results show that GOx encapsulation in a chitosan matrix is capable of providing high catalytic activity for at least six months.

The stability of the biosensors decreased dramatically when they were stored in glucose 2 mM, but prior crosslinking of GOx before its encapsulation into chitosan significantly improved the stability of the biosensors. In addition, we demonstrated that the addition of catalase enhanced and protected the chitosan-based membrane from intoxication by the local accumulation of H_2_O_2_. Overall, our GOx-chitosan-based glucose biosensors could remain functional for up to one month, thereby showing a high potential for industrial developments in continuous glucose monitors.

## Data Availability

The data that support the findings of this study are available from the corresponding authors upon reasonable request.

## Supplementary Materials

The following supporting information can be downloaded at: https://www.mdpi.com/article/10.3390/s23010465/s1, Figure S1: Stability in function of time of the chitosan-based biosensing membranes deposed on gold screen-printed electrodes during continuous measurement of glucose for 24 h. Experimental conditions: PBS(1X) medium, [Glucose] = 0.5 mM (stable), under stirring (200 rpm) and current sampling frequency of 10 s and; Figure S2: Study by cyclic voltammetry at various scan rates between 0–1 V of the chitosan-based biosensing membranes deposed on gold screen-printed electrodes, [Glucose] = 1 mM.
