# Supplementary material for: Investigation of GOx Stability in a Chitosan Matrix: Applications for Enzymatic Electrodes
Source: Sensors (Basel). 2023 Jan 1;23(1):465. doi: 10.3390/s23010465 (PMC9824325; doi:10.3390/s23010465)
Supplement: Supplementary file 1 [file sensors-23-00465-s001.zip › sensors-2074106-supplementary.pdf]

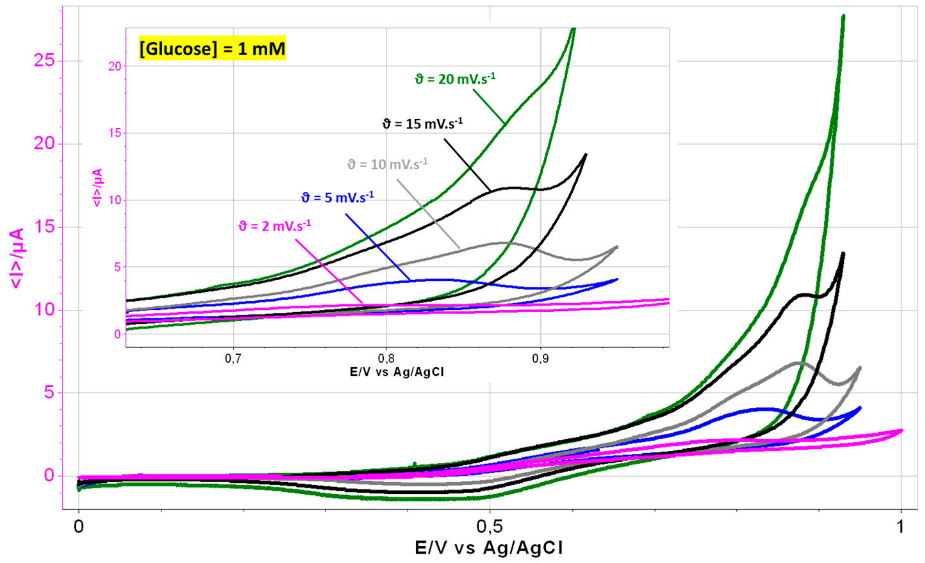

Figure S1. Study by cyclic voltammetry at various scan rates between 0 V – 1 V of the chitosan-based biosensing membranes deposed on gold screen-printed electrodes, [Glucose] = 1 mM.

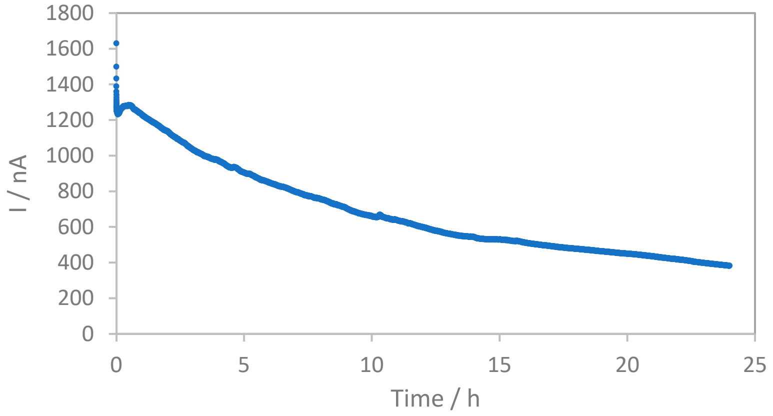

Figure S2. Stability in function of time of the chitosan-based biosensing membranes deposed on gold screen-printed electrodes during continuous measurement of glucose for 24 hours. Experimental conditions: PBS(1X) medium, [Glucose] = 0.5 mM (stable), under stirring (200 rpm) and current sampling frequency of 10 seconds.
